# Professional Perspectives on Mental Distress: Exploring Attributional Differences and Their Association With Stigmatizing Attitudes Among Psychiatrists, Psychologists, and Social Workers

**DOI:** 10.1177/00207640251383193

**Published:** 2025-11-03

**Authors:** Martin Wolgast, Katarina Fredriksson Tham, Maja Straht, Henrik Levinsson

**Affiliations:** 1 Department of Psychology, Lund University, Sweden

**Keywords:** attributional frameworks, stigmatizing attitudes, mental health professionals, biological models, cognitive-behavioral models, social-realist perspectives, interdisciplinary care

## Abstract

**Background:**

Mental health professionals often differ in their explanatory models of mental distress, which may influence their attitudes toward individuals experiencing such conditions. Stigmatizing attitudes among providers can adversely affect therapeutic relationships, service quality, and recovery outcomes.

**Aims:**

This study investigates the attributional frameworks—biological, cognitive-behavioral, psychodynamic, and social-realist—endorsed by psychiatrists, psychologists, and social workers in Swedish adult psychiatry. It further examines how these explanatory preferences are associated with stigmatizing attitudes toward individuals with mental distress.

**Method:**

A cross-sectional survey was conducted with 715 licensed psychiatrists, psychologists, and social workers. Attributional orientations were measured using a revised version of the Maudsley Attitude Questionnaire (MAQ-R), and stigma was assessed with the revised Opening Minds Stigma Scale for Health Care Providers (OMS-HC-R). Group differences were analyzed using General Linear Models, and associations between attributional frameworks and stigma were explored through linear regression. Given the cross-sectional design, causal inferences cannot be drawn from the observed associations.

**Results:**

Psychiatrists reported significantly stronger endorsement of biological attributions compared to psychologists and social workers, while psychologists favored cognitive-behavioral and psychodynamic explanations. Social workers exhibited the strongest preference for social-realist attributions. Endorsement of biological and cognitive-behavioral models was positively associated with higher stigma scores, although these associations may reflect broader occupational or systemic influences. In contrast, social-realist attributions were associated with lower stigma. Psychodynamic attributions showed no significant association.

**Conclusions:**

Differences in attributional frameworks among mental health professionals are associated with variation in reported stigmatizing attitudes. These findings underscore the importance of addressing explanatory diversity in interdisciplinary training and suggest that nuanced understandings of etiology may inform stigma-reduction efforts. Future research should explore the role of contextual and institutional factors, as well as potential confounders, in shaping these relationships.

## Introduction

Mental distress, though often framed in clinical terms, emerges at the intersection of personal, social, and institutional processes. While global health agencies increasingly highlight its public health impact, the dominant approaches to understanding and responding to distress remain heavily influenced by biomedical frameworks. These frameworks, though powerful, tend to foreground individual pathology, frequently overlooking the cultural, political, and structural conditions in which suffering is produced and made legible as “mental illness” (Kirmayer, 2025; Mills, 2022). Addressing stigma in mental health care thus requires not only individual-level interventions but also a critical interrogation of the professional ideologies that shape how distress is conceptualized and treated (Tomičić & Gjorgjioska, 2024).

A critical and often underexplored aspect of this challenge is the stigma attached to mental health problems, which operate at multiple levels and permeates even the institutions designed to provide care (Thornicroft et al., 2007). Whereas stigma in the general population has been widely studied, growing evidence highlights the prevalence of stigma among healthcare professionals, including psychiatric staff, which can have significant consequences for the quality of care and recovery outcomes for individuals with mental health conditions (Mehta et al., 2015). This form of stigma, often referred to as “provider stigma,” manifests through discriminatory attitudes, dismissive behaviors, and inequitable decision-making processes within psychiatric and medical settings (Knaak et al., 2017).

Stigma among psychiatric staff is particularly concerning as these professionals are expected to take an active role in dismantling societal prejudices. Indeed, Schulze (2007) highlights the complex role that professionals play in both perpetuating and experiencing stigma. While some professionals do endorse stereotyped views, they are also subject to stigmatizing representations and institutional dynamics. This dual position invites a more nuanced understanding of stigma, not as a static trait of the professional, but as a relational and systemic phenomenon embedded in psychiatric institutions. Given this understanding, these attitudes may be shaped by a combination of factors, including stress, exposure to severe cases of mental distress, or an overemphasis on explanations for mental distress that frame it in terms of mental health conditions that is chronic and immutable (Corrigan et al., 2009). For example, studies have found that psychiatric staff are more likely to attribute behaviors of patients with schizophrenia to personal characteristics rather than situational factors, reinforcing stereotypes of unpredictability and chronicity (Angermeyer and Dietrich, 2006). Such stigma might significantly affect the therapeutic alliance between patients and providers, a critical component of effective psychiatric care (Corrigan & Watson, 2002). Dismissive or paternalistic attitudes can erode trust and reduce patient engagement, ultimately hindering treatment adherence and recovery (Link & Phelan, 2001).

The consequences of staff stigma extend beyond patient care, influencing workplace culture and professional dynamics. Psychiatric staff who internalize stigmatizing attitudes may experience moral distress or burnout, further compromising the quality of care (Corrigan et al., 2009). Additionally, trainees entering psychiatric professions may inadvertently adopt these attitudes, perpetuating a cycle of stigma within the field (Schulze, 2007). Addressing stigma among psychiatric staff requires targeted interventions, including structured anti-stigma training, reflective practice, and systemic reforms that promote holistic and person-centered care models.

While stigmatizing attitudes among psychiatric staff is a critical issue, it operates within a broader societal context where mental health stigma remains deeply entrenched. Societal stigma manifests through labeling, stereotyping, and discrimination against individuals with mental health problems (Hinshaw, 2007). These stereotypes, such as beliefs that individuals with mental health problems are dangerous or incapable of contributing to society, perpetuate social exclusion and limit access to opportunities in employment, education, and healthcare (Thornicroft et al., 2009). This dual dynamic - societal stigma reinforcing provider stigma - creates additional barriers for individuals seeking mental health support, as they may fear judgment not only from society but also from the professionals tasked with their care (Schulze & Angermeyer, 2003).

Efforts to combat stigma must therefore address both societal attitudes and the specific challenges within psychiatric practice. Programs such as Time to Change in the United Kingdom and WHO’s Mental Health Action Plan 2013 to 2030 emphasize the need for systemic interventions, including professional education and policy reforms that promote parity between mental and physical health (Henderson & Thornicroft, 2013; World Health Organization, 2021). Effective strategies include stigma-reduction training for psychiatric staff, which has been shown to increase empathy and reduce prejudices, and organizational changes that encourage interdisciplinary collaboration (Mehta et al., 2015).

In this context, it is important to notice that mental health professionals are not neutral observers of mental distress; their interpretations are shaped by the epistemic frameworks embedded in their training, practice environments, and institutional affiliations (Harland et al., 2009; Honkasilta & Koutsoklenis, 2022). Psychiatrists, psychologists, and social workers are socialized into at least partially distinct modes of understanding and responding to mental health problems. These frameworks in turn influence not only clinical decision-making but also how professionals relate to service users, assess risk, and navigate ethical tensions between care and control (Broome, 2006; Kirmayer, 2025). Indeed, the ways in which mental distress and suffering are conceptualized have been suggested to be a central factor in shaping professional attitudes and behaviors in psychiatric settings (Boyle, 2013). Hence, analyzing the dynamics of different conceptualizations of mental distress is of importance to understanding how stigma may be perpetuated or challenged within different sectors of mental health care.

A central part of different conceptualizations of mental distress are differences in attributional frameworks, which refer to the explanatory models professionals use to understand the causes and nature of distress (Pilgrim, 2015). Attributional frameworks are typically grouped into biological, psychological, and social perspectives, each reflecting distinct paradigms for interpreting mental health conditions (Pilgrim, 2015). Importantly, the frameworks that professionals adopt are influenced by their disciplinary training, theoretical orientation, and clinical experience, and they play a critical role in shaping stigma within healthcare contexts (Honkasilta & Koutsoklenis, 2022). In the paragraphs below, we describe and discuss the attributional frameworks explored in the present study.

### Biological Perspectives

The biological framework, which emphasizes genetic predispositions, neurochemical imbalances, and brain abnormalities, has long held epistemic dominance in psychiatric practice (Middleton & Moncrieff, 2019). This orientation is embedded within the broader biomedical model, which conceptualizes mental distress as a disorder of the brain, typically requiring medical interventions such as psychotropic medication or electroconvulsive therapy (Kawa & Giordano, 2012). Far from being a neutral scientific lens, the biomedical model functions as a historically situated and institutionally sanctioned discourse, operationalized through diagnostic systems like the DSM that pathologize subjective suffering and align clinical legitimacy with presumed biological causation (Horwitz, 2007; Kawa & Giordano, 2012).

While biological explanations can mitigate individual blame, for instance by reframing conditions like schizophrenia as illnesses rather than moral failings (Corrigan et al., 2006; Haslam & Kvaale, 2015), they also have documented negative consequences for how individuals are perceived and treated and some empirical studies have indicated that biological attributions are associated with heightened perceptions of dangerousness, chronicity, and unpredictability (Angermeyer et al., 2014; Haslam, 2011; Kvaale et al., 2013). Furthermore, these representations can legitimize coercive interventions, such as involuntary hospitalization or forced medication, by constructing service users as incapable of rational participation in treatment decisions (Angermeyer et al., 2014; Pescosolido et al., 2010; Read et al., 2006).

### Psychological Perspectives

Psychological frameworks encompass a range of approaches to understanding mental distress, with significant distinctions between cognitive-behavioral and psychodynamic perspectives (Burbridge-James & Iwanowicz, 2018). These frameworks focus on individual agency, cognitive processes, and personal experiences as key contributors to mental health conditions. Both perspectives are influential within the field of psychology, though they differ in their theoretical underpinnings and explanatory models (Wampold, 2015).

Cognitive-behavioral frameworks emphasize the role of learned behaviors, thought patterns, and individual agency in the development and persistence of mental health conditions (Beck & Haigh, 2014). These perspectives form the foundation of therapeutic approaches such as cognitive-behavioral therapy (CBT), which aims to identify and modify maladaptive thoughts and behaviors that contribute to distress (Reiss et al., 2019). Cognitive-behavioral attributions often highlight recovery potential by focusing on actionable strategies for change, fostering a sense of empowerment among patients (Hofmann et al., 2012). However, these attributions may inadvertently contribute to stigmatizing attitudes by placing responsibility for recovery primarily on the individual. For instance, a psychologist might attribute a lack of progress in therapy to personal failure or non-compliance, overlooking systemic barriers or external stressors that hinder recovery (Lundberg et al., 2008). This focus on individual responsibility can lead to tension when cognitive-behavioral professionals work alongside others who prioritize systemic or biological explanations.

Psychodynamic frameworks, in contrast, emphasize the role of unconscious conflicts, early relational experiences, and internal emotional struggles as central to understanding mental distress (Gabbard, 2017). These perspectives are foundational to psychodynamic therapy, which seeks to uncover and address unresolved emotional conflicts and patterns that contribute to an individual’s suffering (Lemma, 2016). Psychodynamic attributions provide a nuanced understanding of the underlying causes of distress, often fostering empathy and deeper insight into a patient’s experiences (Reiss et al., 2019). Despite their emphasis on understanding and empathy, psychodynamic attributions may inadvertently reinforce stigma by framing distress as rooted in internal deficiencies or unresolved psychological issues. For example, a professional relying heavily on psychodynamic explanations might perceive certain behaviors or patterns as reflective of inherent vulnerabilities, potentially leading to biases or stereotypes about the individual’s ability to recover.

### Social Perspectives

Social frameworks situate mental health conditions within broader systemic and structural contexts, emphasizing factors such as socioeconomic inequality, discrimination, and adverse childhood experiences (Kirkbride et al., 2024). Social workers, in particular, are trained to adopt this perspective, often focusing on advocacy, community-based interventions, and structural change as critical components of mental health care (Bland et al., 2021). From this perspective, one might attribute a patient’s depression to unemployment, housing insecurity, or systemic racism, emphasizing the need for external, systemic solutions rather than individual-level interventions (Kirkbride et al., 2024).

Social attributions can mitigate stigma by reducing blame and emphasizing the broader societal conditions that contribute to mental distress (Pescosolido et al., 2010). However, they can also oversimplify the complexity of mental health conditions by underemphasizing individual and biological factors (Thoits, 2011). Furthermore, professionals who prioritize social attributions may face challenges when working in systems dominated by biomedical models, leading to potential misalignment in care approaches and patient outcomes (Thoits, 2011).

### Connections Between Frameworks, Professions, and Stigma

A central assumption underlying the present study is that the disciplinary training of different psychiatric professionals significantly shapes their reliance on specific attributional frameworks, which in turn influences their attitudes toward mental distress and psychiatric patients. For example, psychiatrists, who are predominantly trained within the medical model, are likely to emphasize biological explanations. As discussed above, this can lead to stigmatizing beliefs rooted in perceptions of chronicity and patient unpredictability, as well as a focus on pharmacological solutions at the expense of psychosocial care. Psychologists on the other hand, while emphasizing recovery and agency, may inadvertently promote stigma by overemphasizing individual responsibility for mental distress, particularly when patients do not meet therapeutic goals. Social workers, by focusing on structural factors, may foster empathy and reduce blame but risk neglecting the interplay of biological and psychological dimensions in patient care (Kirkbride et al., 2024). In sum, differences in how mental health professionals conceptualize mental distress may shape, and be shaped by, their clinical experiences, institutional contexts, and broader cultural assumptions. While explanatory frameworks have been associated with varying levels of stigmatizing attitudes, the nature of this relationship remains contested and likely bidirectional.

Research highlights that these professional differences in attributional frameworks are associated with varying levels and forms of stigma. Angermeyer et al. (2011) found that those who endorsed strong biological attributions were more likely to view patients as dangerous and support coercive measures, whereas those emphasizing psychological attributions were more likely to exhibit frustration or disappointment with perceived “non-compliance.” Similarly, those adopting social frameworks often displayed the lowest levels of stigma but sometimes lacked practical strategies for addressing the immediate needs of individuals within structurally inequitable systems (Thoits, 2011). These findings underscore the importance of understanding how disciplinary orientations shape professional attitudes and their impact on stigma. Differences in attributional frameworks are not merely academic; they have tangible consequences for how patients are treated, the types of interventions prioritized, and the overall experience of care (Corrigan et al., 2014). For example, a patient with a dual diagnosis of schizophrenia and homelessness might encounter vastly different responses from a psychiatrist focused on medication adherence, a psychologist emphasizing therapy, and a social worker advocating for housing solutions. While these approaches can be complementary, differences in attributional priorities can also lead to fragmented care and reinforce stigmatizing attitudes among professionals, particularly when collaboration is limited or strained (Schulze, 2007).

In summary, attributional frameworks are central to how professionals conceptualize mental distress, and these frameworks are likely to be influenced by disciplinary training and professional identity. In addition, differences in attributional perspectives among psychiatrists, psychologists, and social workers is likely to be associated with variations in stigmatizing attitudes, shaping how patients are perceived. Understanding these dynamics is critical for addressing stigma and fostering more equitable and compassionate care environments.

While attributional frameworks have been widely studied in relation to stigma in general populations, the systematic examination of how different psychiatric professions conceptualize mental distress remains underexplored. Existing research has identified variations in the adoption of biological, psychological, and social frameworks, yet little is known about how these differences align with specific professional roles in psychiatry, psychology, and social work. Even less is understood about the relationship between these frameworks and stigmatizing attitudes in relation to individuals seeking mental health care.

Another underexamined area is the specific association between attributional frameworks and stigmatizing attitudes. While it is recognized that biological attributions can reduce personal blame but increase perceptions of chronicity, and that psychological or social attributions may foster either empathy or misplaced responsibility, most studies have treated these factors in isolation (e.g. Angermeyer & Dietrich, 2006; Haslam & Kvaale et al., 2015). In addition, there is a lack of research regarding how different attributional frameworks are associated with stigmatizing beliefs in professional settings. This limitation has left a critical gap in understanding how stigma operates in the very systems designed to treat mental distress. Furthermore, differences in how mental health professionals conceptualize mental distress may shape, and be shaped by, their clinical experiences, institutional contexts, and broader cultural assumptions. While explanatory frameworks have been associated with varying levels of stigmatizing attitudes, the nature of this relationship remains contested and likely bidirectional.

In sum, while research has explored public stigma, less attention has been paid to how mental health professionals themselves reproduce or resist stigmatizing beliefs. When studied, such attitudes are often treated as individual biases rather than reflections of professional training, institutional ideology, or epistemic positioning (Kirmayer, 2025; Schulze, 2007). Moreover, few studies have directly examined how professionals’ explanatory preferences relate to stigma, particularly across professions. This represents a critical gap, as attributional models are not merely descriptive tools, but part of a wider apparatus of meaning-making and governance in mental health care (Honkasilta & Koutsoklenis, 2022; Tomičić & Gjorgjioska, 2024). The present study seeks to address this critical gap by exploring two key questions: whether attributions of mental distress differ among psychiatric professions and how these differences correlate with stigmatizing attitudes among practicing clinical staff. In doing so, rather than treating stigma as an individual bias or professional deficit, we approach it as a phenomenon shaped by the interaction between epistemic frameworks and institutional practice (Schulze, 2007; Tomičić & Gjorgjioska, 2024). Importantly however, we do not claim causal relationships between explanatory orientation and stigma; rather, we explore how these orientations may reflect and reinforce broader ideologies of mental health care. In doing so, we contribute to a growing body of critical research that interrogates the normative underpinnings of psychiatric knowledge production and its implications for ethical, person-centered care (Honkasilta & Koutsoklenis, 2022; Kirmayer, 2025).

## Purpose and Hypotheses

The purpose of this study was to investigate how psychiatric professions - psychiatrists, psychologists, and social workers - differ in their attributional frameworks for understanding psychological and emotional distress and how these differences relate to general levels of stigmatizing attitudes. As described above, whereas attributional frameworks have been explored in general populations, limited research has examined how these frameworks vary across professions and how specific attributions are associated with stigmatizing attitudes among practicing clinicians.

More specifically, the present study seeks to achieve the following objectives:

1. Identify and compare how psychiatrists, psychologists, and social workers prioritize biological, cognitive-behavioral, psychodynamic, and social attributions for mental distress.
2. Examine the relationship between reliance on specific attributional frameworks and general levels of stigmatizing beliefs among professionals.

By addressing these objectives, the study aims to advance understanding of how disciplinary orientations influence stigma within mental health care and provide insights into the dynamics of stigma in professional settings.

### Hypotheses

The study was designed to test the following hypotheses:

1. Differences in attributional frameworks across professions:
  H1: Psychiatrists will score higher on biological attributions compared to psychologists and social workers.
  H2: Psychologists will score higher on cognitive-behavioral and psychodynamic attributions compared to psychiatrists and social workers.
  H3: Social workers will score higher on social attributions compared to psychiatrists and psychologists.
2. Attributional frameworks and general levels of stigmatizing attitudes:
  H4: Higher reliance on biological attributions will be associated with higher general levels of stigmatizing attitudes.
  H5: Higher reliance on cognitive-behavioral attributions will be associated with moderate levels of stigmatizing attitudes.
  H6: Higher reliance on psychodynamic attributions will be associated with moderate levels of stigmatizing attitudes.
  H7: Higher reliance on social attributions will be associated with lower general levels of stigmatizing attitudes.

## Materials & Methods

### Participants and Procedure

E-mails were sent to medical professionals in Swedish adult psychiatry. Staff in other areas of psychiatry, such as child and adolescent psychiatry, were not included in this study. Access to e-mail addresses was given through e-mail correspondence with the registry clerk of different regions in Sweden. The response rate of the survey was 22%. All data collection was anonymized, and no information was collected that made it possible to indirectly or directly identify specific respondents. All respondents gave their informed consent prior to participation. For the present study, only respondents that stated that their profession was psychiatrist, psychologist or social worker were included in the sample (*n* = 715). Demographic statistics of the sample are presented in Table 1.

**Table 1.** Demographic Data of the Sample (*n* = 715).

Background variable	*n*	*M* (*SD*)
Gender		
Female	452	
Male	250	
Other/missing	13	
Age		36.6 (12.0)
Profession		
Psychiatrist	258	
Psychologist	273	
Social worker	184	
Years worked in psychiatry		10.5 (10.0)
Form of care		
Outpatient care	548	
Inpatient care	159	
Intermediary care	8	
Primary patient populations		
Affective disorders	480	
Personality disorders	444	
Neuropsychiatric disorders	416	
Psychotic disorders	254	
Trauma and stress-related disorders	360	
Substance-related and addictive disorders	246	
Eating disorders	102	
Gender dysphoria	65	
Other	44	

### Measures and Instruments

#### Revised Version of the Opening Minds Stigma Scale for Health Care Providers (OMS-HC-R)

The OMS-HC is a self-assessment instrument consisting of 20 questions, developed by Kassam et al. (2012). The scale aims to measure healthcare providers’ stigmatizing attitudes and behaviors toward individuals with mental illness. The measure is comprised by statements regarding patients with psychiatric diagnoses (e.g. *“Despite my professional beliefs, I have negative reactions toward people who have mental illness,” “I would see myself as weak if I had a mental illness and could not fix it myself”*, *“More than half of people with mental illness don’t try hard enough to get better.”* and *“There is little I can do to help people with mental illness”*) to which the respondents are asked to rate the extent to which they agree/disagree on a five-point scale.

The scale has been condensed to 15 questions (Modgill et al., 2014), and it was this revised version which was used in the present study. Modgill et al. (2014) reported a high level of internal consistency for the 15-item scale (*α* = .79) in a sample of general health care providers. In the present study, the scale showed similar levels of internal (*α* = .75).

#### Revised Version of Maudsley Attitude Questionnaire (MAQ-R)

The MAQ is a self-report instrument designed to identify conceptual explanatory models among professionals in psychiatric healthcare (Harland et al., 2009). Originally consisting of 128 items, the MAQ explores participants’ ontological assumptions about mental illness through statements grouped into eight paradigms: biological, cognitive, behavioral, psychodynamic, social-realist, social-constructivist, nihilistic, and spiritual. Participants evaluate these paradigms across four psychiatric diagnoses - schizophrenia, generalized anxiety disorder (GAD), major depressive disorder (MDD), and antisocial personality disorder (APD) - using a 5-point scale from 1 (“Strongly disagree”) to 5 (“Strongly agree”).

In prior research, MAQ has been used to examine attitudes among future psychiatrists and clinical psychologists, showing significant differences between professions (Harland et al., 2009; Read et al., 2017). In the present study, the focus was narrowed to four explanatory models deemed most relevant to the Swedish psychiatric context in which the study was performed: biological, cognitive-behavioral, psychodynamic, and social-realist. In the revised version used in the present study, “mental illness” was used as a unified concept rather than focusing on specific diagnoses, simplifying the questionnaire to 16 items (4 items per explanatory model), which were translated into Swedish by the study authors using a translation-backtranslation procedure.

The biological model emphasizes brain and nervous system dysfunction, exemplified by statements like “Treatment of mental illness should target underlying biological abnormalities” and “Mental illnesses result from brain dysfunctions.”

The cognitive-behavioral model attributes mental illness to dysfunctional thought processes and maladaptive learned behaviors, represented by statements like *“Mental illness should be treated by challenging and restructuring distorted thoughts and beliefs.”* and “*Mental illness results from maladapted associative learning.*”

The psychodynamic model focuses on unconscious internal conflicts often originating in early life, with statements like *“Treating mental illness involves resolving disturbed early relationships.”* and *“The disorder results from the failure to successfully complete developmental psychic stages”*.

The social-realist model underscores external factors such as societal injustice, poor housing, and unemployment, illustrated by statements like “*The primary causes of mental illness are social factors such as prejudice, inadequate housing, and unemployment.”* and “*Mental illness arises as a consequence of social circumstances or conditions.”*

In the present study, the four subscales all showed adequate levels of internal consistency (Biological: *α* = .76; Cognitive-behavioral: *α* = .74; Psychodynamic: *α* = .79; Social-realist: *α* = .72).

### Data Analysis

Prior to running the analyses, the data was checked for outliers and violations of the assumptions for General Linear Model and linear regression analyses. All assumptions for using the General Linear Model and linear regression were assessed to be met. The relationship between variables was linear, residuals were independent and showed homoscedasticity. Residuals followed an approximately normal distribution. Variance Inflation Factors indicated no multicollinearity, and checks for outliers revealed no significant outliers.

The hypotheses regarding differences between professions in attributional frameworks were examined using General Linear Model with the different attributional models (biological, cognitive-behavioral, psychodynamic and social-realist) as the within-subject factor and profession (psychiatrist, psychologist, social worker/counselor) as the between-subject factor. Here, the omnibus test of the attribution x profession interaction was of interest for the hypotheses, as well as the pairwise comparisons of the estimated marginal for the interaction between the within- and between-subject factors. All analyses were corrected for multiple comparisons using Šidák correction.

The hypotheses regarding the association between the different attributional frameworks and stigmatizing attitudes were examined using linear regression analysis with stigmatizing attitudes as the criterion variable and the attributional frameworks as predictor variables.

### Ethics

The authors assert that all procedures contributing to this work comply with the ethical standards of the relevant national and institutional committees on human experimentation and with the Helsinki Declaration of 1975, as revised in 2008. The study has been performed in full accordance with the Swedish Ethical Review Act and all procedures involving human subjects/patients were approved by the ethics committee at the [MASKED FOR REVIEW PUROPSES]. Written informed consent was obtained from all participants, and anonymity was guaranteed through the Qualtrics survey tool.

## Results

### Differences in Attributional Frameworks Across Professions

As described under “Data analysis” above, the hypotheses regarding differences in attributional frameworks across professions were examined using a mixed model GLM analysis. Descriptive statistics of the scores on the four subscales of the MAQ-R across the different professions are presented in Table 2.

**Table 2.** Descriptive Statistics Data of Scores on the Attributional Frameworks (MOQ-R) Across Professions.

	Biological	Cognitive-behavioral	Psychodynamic	Social-realist
Profession	*M* (*SD*)	*M* (*SD*)	*M* (*SD*)	*M* (*SD*)
Psychiatrist	4.00 (0.53)	3.07 (0.71)	2.57 (0.80)	3.13 (0.66)
Psychologist	3.13 (0.66)	3.35 (0.64)	2.86 (0.86)	3.38 (0.57)
Social worker	3.27 (0.57)	3.05 (0.60)	2.95 (0.83)	3.49 (0.61)

Repeated measures ANOVA revealed a significant multivariate omnibus effect of Profession × Attribution (*F* (6, 1170) = 49.05; *p* < .001; part. *ƞ*^2^ = 0.20) indicating, as hypothesized, a general difference between the professions on the average scores across the different attributional frameworks. Šidák corrected pairwise comparisons indicated that, for the subscale on the MAQ-S measuring Biological attributions, psychiatrist scored significantly higher than psychologists (*M*_D_ = 0.88; *SE* = 0.06; *p* < .001) and social workers (*M*_D_ = 0.73; *SE* = 0.06; *p* < .001), whereas the difference between psychologists and social workers was non-significant (*M*_D_ = −0.14; *SE* = 0.06; *p* = .06). For Cognitive-behavioral attributions, psychologists scored significantly higher than psychiatrists (*M*_D_ = 0.29; *SE* = 0.06; *p* < .001) and social workers (*M*_D_ = 0.30; *SE* = 0.07; *p* < .001), whereas the difference between psychiatrists and social workers was non-significant (*M*_D_ = −0.14; *SE* = 0.06; *p* = .06). When it came to Psychodynamic attributions, both psychologists (*M*_D_ = 0.30; *SE* = 0.06; *p* < .001) and social workers (*M*_D_ = 0.38; *SE* = 0.09; *p* < .001) scored significantly higher than psychiatrists, but the difference between psychologists and social workers (*M*_D_ = −0.08; *SE* = 0.09; *p* = .71) was non-significant. Lastly, for social-realist attributions, social workers (*M*_D_ = 0.37; *SE* = 0.07; *p* < .001) and psychologists (*M*_D_ = 0.25; *SE* = 0.06; *p* < .001) scored higher than psychiatrists. However, there were no significant difference between social workers and psychologists (*M*_D_ = 0.11; *SE* = 0.07; *p* *=* .23). Hence, the results in provided partial support for the hypotheses under investigation: there were significant differences between the professions in relation to the attributional frameworks: Psychiatrists showed the strongest support for biological attributions and psychologists for cognitive-behavioral attributions, providing support for hypotheses 1 and 2. For both psychodynamic and social-realist attributions, both psychologists and social workers had significantly higher scores than psychiatrists, but there was no significant difference between psychologists and social workers, hence providing partial support for hypotheses 2 and 3.

### Attributional Frameworks and General Levels of Stigmatizing Attitudes

The hypotheses regarding associations between the attributional frameworks and stigmatizing attitudes were examined using linear regression analyses. Results are presented in Table 3.

**Table 3.** Results of linear regression analysis of associations between attributional framework and stigmatizing attitudes.

Framework	*b* (*SE)*	*β*	*t*	*p*
Biological	1.16 (1.48)	.182	4.30	<.001
Cognitive-behavioral	0.67 (0.31)	.097	2.14	.033
Psychodynamic	0.34 (0.25)	.063	1.37	.171
Social-realist	−1.06 (0.34)	−.147	−3.15	.002

As can be seen in Table 3, the results indicated that both Biological and Cognitive-behavioral attributions were positively associated with stigmatizing attitudes toward patients with psychiatric diagnoses, where Biological attributions was the strongest positive predictor. In addition, Social-realist attributions were negatively associated with stigmatizing attitudes whereas the positive association between Psychodynamic attributions and stigmatizing attitudes was non-significant. Hence, the performed analyses generally supported the hypotheses under investigation (H4–7), except for the fact that there was no significant association between Psychodynamic attributions and stigmatizing attitudes.

## Discussion

The performed study provides a novel contribution to the understanding of professional attitudes toward mental distress by systematically exploring how attributional frameworks relate to stigmatizing beliefs among practicing clinicians. While previous research has primarily focused on public attitudes or student samples, this investigation uniquely examines these dynamics within a diverse group of practicing psychiatrists, psychologists, and social workers. The findings demonstrate significant differences in how these professions attribute mental distress and the degree to which these attributions are related to stigmatizing attitudes. These insights offer a deeper understanding of how professional roles and training shape clinical perspectives and contribute to the persistence of stigma within mental health systems.

### Attributional Frameworks Across Professions

The distinct attributional preferences identified in this study reflect the professional training and theoretical foundations specific to psychiatrists, psychologists, and social workers. Psychiatrists’ stronger alignment with biological attributions likely mirrors their exposure to frameworks emphasizing neurological and genetic explanations, as supported by diagnostic systems such as the DSM (Kawa & Giordano, 2012). While these attributions can depersonalize mental distress by removing individual blame, they also risk perpetuating stigma by framing distress as static and immutable (Kvaale et al., 2013). In contrast, psychologists predominantly endorsed cognitive-behavioral and psychodynamic frameworks, underscoring their focus on individual agency, thought processes, and internal dynamics. These perspectives, while offering empowering narratives of change, may inadvertently shift responsibility for recovery onto individuals, potentially overlooking broader systemic barriers (Lundberg et al., 2008). Social workers’ reliance on social-realist attributions highlights their emphasis on structural factors such as inequality and marginalization, fostering an understanding of mental distress as deeply embedded within social contexts (Kirkbride et al., 2024).

These differences underscore the varied ways professionals interpret mental distress, revealing the potential for both complementary strengths and tensions when addressing stigma within interdisciplinary mental health systems. Notably, this study’s focus on practicing clinicians provides critical real-world insights into how these attributional frameworks operate in professional contexts, moving beyond theoretical or academic discourse.

### Stigmatizing Attitudes and Their Associations With Attributions

Our findings suggest patterned associations between explanatory orientations and reported stigma among mental health professionals. Endorsement of biological attributions was associated with higher stigma scores, echoing prior research that links biogenetic framings to narratives of chronicity, unpredictability, and risk (Haslam & Kvaale, 2015). From a critical perspective, these associations should not be understood as evidence that biology itself produces stigma, but rather as a reflection of how the biomedical model has been institutionalized within psychiatric practice. When distress is framed primarily through neurobiological dysfunction, service users may be construed as less capable of recovery or autonomous decision-making, thereby legitimizing paternalistic and coercive interventions (Angermeyer et al., 2014; Read et al., 2006).

By contrast, social-realist attributions were associated with lower stigma, likely because they situate distress within structural and relational conditions rather than reducing it to individual pathology (Kirmayer, 2025; Pescosolido et al., 2010). Such perspectives foreground the social determinants of mental health and open possibilities for solidarity-based, contextualized responses that resist pathologization. Interestingly, psychodynamic attributions did not show a significant association with stigma in this sample. Their interpretive emphasis on unconscious conflict and relational meaning may neither directly reinforce nor systematically counteract stigmatizing attitudes, suggesting that their implications for stigma are more ambivalent or context-dependent.

Taken together, these findings underscore that attributional frameworks are not neutral explanatory devices, but carry epistemic and political consequences for how professionals relate to individuals in distress. The variability observed here reinforces the importance of fostering professional environments that encourage reflexive engagement with dominant paradigms, and that legitimize diverse perspectives, particularly those that challenge reductionist or deterministic narratives. Such epistemic pluralism may represent a facilitating condition for reducing stigma within mental health care.

### Possible Implications for Practice and Training

By examining attributional frameworks and stigma in practicing clinicians, this study offers actionable insights into professional attitudes toward mental distress. Addressing stigma within mental health care requires a critical evaluation of how professional training perpetuates certain explanatory models at the expense of others. For instance, training programs for psychiatrists may need to move beyond emphasizing biomedical frameworks to include greater engagement with systemic and experiential understandings of distress. Similarly, psychologists and social workers might benefit from enhanced exposure to alternative explanatory models that promote shared understanding and reduce fragmentation in care approaches.

In practice, these findings suggest that interventions to reduce stigma among professionals should be tailored to the attributional biases of specific disciplines. For example, anti-stigma programs targeting psychiatrists might focus on reframing notions of chronicity, while those aimed at psychologists could emphasize the broader systemic and relational contexts of distress. Social workers, often positioned to challenge stigma through advocacy and systemic interventions, could further benefit from opportunities to integrate individual experiences within their structural analyses.

### Strengths, Limitations and Directions for Future Research

A central strength of this study lies in its focus on practicing clinicians, which bridges a persistent gap between theoretical research on stigma and its enactment in everyday psychiatric practice. By examining psychiatrists, psychologists, and social workers together, the study provides a rare comparative snapshot of how professional knowledge systems align with attitudes toward mental distress.

At the same time, several limitations must be acknowledged. First, reliance on self-reported survey data raises the possibility of social desirability bias, whereby participants may underreport stigmatizing beliefs. Second, while the OMS-HC-R provides a standardized tool for measuring provider stigma, its items do not exclusively capture prejudice. Some responses may instead reflect broader affective or structural dynamics: for example, reduced compassion may signal burnout, and reluctance to seek care may stem from distrust of psychiatric institutions or fear of professional repercussions. This interpretive ambiguity highlights the need for more refined instruments that can distinguish attitudinal stigma from occupational strain or systemic critique. Third, the cross-sectional design prevents causal inference. Associations between explanatory orientations and stigma may reflect reciprocal influences or shared contextual drivers. Relatedly, our study did not measure potential confounders such as clinical setting, patient population, years of experience, or prior exposure to anti-stigma training. These factors may shape both explanatory frameworks and stigma, and should be incorporated into future analyses. Finally, as the sample was obtained through convenience recruitment, generalizability to all psychiatric professionals is limited.

These limitations point to important directions for future research. Longitudinal and mixed-methods designs are needed to explore how explanatory orientations and stigma co-evolve over time, particularly in relation to changes in professional training, institutional cultures, and broader systemic reforms. Cross-national studies would allow comparison of how attributional frameworks and stigma interact with diverse cultural and policy contexts, while qualitative work could unpack how clinicians negotiate these frameworks in practice. More broadly, future research should move beyond attitudinal surveys to interrogate the institutional and epistemic conditions under which stigma is reproduced or resisted within mental health systems.

## Conclusion

This study underscores the complexity of how mental health professionals conceptualize distress, and how these explanatory orientations are associated with stigmatizing attitudes. The differences observed between psychiatrists, psychologists, and social workers are not merely disciplinary variations, but reflections of broader epistemic and institutional formations within psychiatry and allied fields. Understanding stigma in this context requires moving beyond individual attitudes to interrogate the dominant narratives and professional ideologies that structure mental health care.

Rather than prescribing simple stigma-reduction interventions, our findings call for sustained critical dialog about the knowledge systems that shape clinical practice, the values embedded within them, and the institutional conditions under which they are enacted. Fostering epistemic pluralism, where social, psychological, and biological perspectives coexist without hierarchical reduction, may be a necessary condition for creating more equitable and humane forms of care. Ultimately, challenging stigma requires not only addressing prejudicial beliefs, but also scrutinizing the conceptual and institutional frameworks that reproduce them.
